# Determination of 48 elements in 7 plant CRMs by ICP-MS/MS with a focus on technology-critical elements

**DOI:** 10.1007/s00216-022-04497-3

**Published:** 2023-01-10

**Authors:** Simone Trimmel, Thomas C. Meisel, Shaun T. Lancaster, Thomas Prohaska, Johanna Irrgeher

**Affiliations:** Montanuniversität Leoben, Leoben, Austria

**Keywords:** Plant reference materials, Microwave digestion, Rare-earth elements, N_2_O, Collision/reaction cell

## Abstract

Seven plant certified reference materials (NIST SRM1515 Apple Leaves, NIST SRM1547 Peach Leaves, BCR-129 Hay Powder, BCR-670 Aquatic Plant, GBW07603 Bush Twigs and Leaves, GBW10015 Spinach Leaves and NCS ZC73036a Green Tea) were analysed for their mass fractions of 48 elements by inductively coupled plasma tandem-mass spectrometry (ICP-MS/MS): Li, Be, Na, Mg, Al, Ca, V, Cr, Mn, Fe, Co, Ni, Cu, Zn, Ga, Ge, As, Se, Rb, Sr, Y, Nb, Mo, Ag, Cd, Sb, Te, Ba, La, Ce, Pr, Nd, Sm, Eu, Gd, Tb, Dy, Ho, Er, Tm, Yb, Lu, Ta, Tl, Pb, Bi, Th, U. Special focus was put on the determination of technology-critical elements (TCEs), to which, e.g. Li, Be, Ga, Ge, Nb, Sb, Ta, Tl, Bi, and the rare-earth elements (REEs, lanthanides and Y) are counted. Closed-vessel microwave digestion was performed using HNO_3_, H_2_O_2_ and HBF_4_. The average bias for certified values is  − 1% ± 13% (*SD*). Limits of detection (*x*_L_) in the measured solutions lie between 13 fg g^−1^ (Tb) and 52 ng g^−1^ (Ca). This article seeks to provide an optimised measurement procedure for the determination of element mass fractions of emerging importance in environmental samples, which are challenging to analyse with more traditional techniques such as single-quad ICP-MS. In addition, it aims to improve the characterisation of commonly used plant reference materials by providing mass fraction data for rarely studied elements.

**Graphical abstract:**

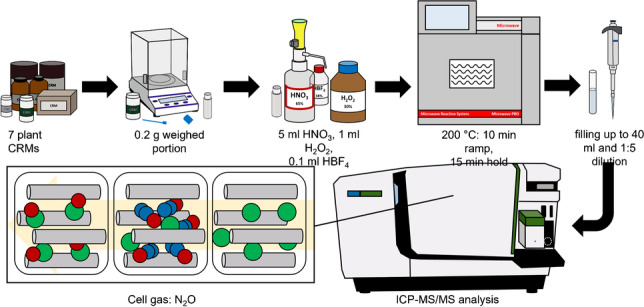

**Supplementary Information:**

The online version contains supplementary material available at 10.1007/s00216-022-04497-3.

## Introduction

Technology-critical elements (TCEs) are a non-uniformly defined group of elements which are deemed critical for modern technology and our societies due to their limited availability in relation to global demand [1]. They all have in common that they serve very specific uses in novel technologies. Their peculiar chemical properties are not fully investigated yet and keep opening new doors in the development of high-tech applications across various fields, such as information and telecommunication technology, renewable energy, semiconductors, speciality alloys or catalysts [2–4]. The downside of the vast technological applicability of these valuable elements is our rapidly increasing dependence on them, as it is not or hardly possible to replace them in many uses. In the last couple of decades, the use of some elements has increased by several orders of magnitude, with the demand still rising [5].

Due to the strongly increased use of TCEs during the last decades, increased dispensation to the environment can be observed during mining, processing, manufacturing usage, disposal and recycling. Therefore, increased levels can be suspected to be present in water, soil, plants, wildlife and humans. Relatively little is known both about the exact amounts present, material flows and possible environmental impacts [1]. The two main reasons for the scarce environmental TCE data are the very short time lapse in which they gained economic importance and the challenging analytical determination of several of these elements, especially with the extremely low levels found in environmental samples [6].

The most commonly used method for multi-element analysis of trace elements is inductively coupled plasma mass spectrometry (ICP-MS). Traditional single-quadrupole instruments, which are popular due to high throughput and low costs, are limited by spectral interferences. To some extent, they can be overcome by the use of collision/reaction cell technology (CRC), which was introduced in the late 1980s. Collisions of interferences with an inert gas (typically He) lead to their loss in kinetic energy, which prevents them from reaching the detector. Reaction gases, in turn, are used to alter the chemical nature of either the analyte or the interference. A common example is the reaction of an analyte with an oxidising gas such as O_2_ by which its mass-to-charge (*m*/*z*) ratio can be shifted to a less strongly interfered *m*/*z* ratio [7]. Another option to deal with interferences would be sector field ICP-MS, which shines with excellent mass resolution. Its drawbacks are a large footprint and longer measurement cycles, which would make the measurement of large sample sets resource-intensive [8]. The introduction of ICP-tandem mass spectrometry (ICP-MS/MS) in 2012 led to a significant extension of the possibilities of quadrupole-based instruments, giving additional control about the ions entering the cell and thus allowing for more effective interference-removal [9]. Its applicability with O_2_ as reaction gas has been demonstrated also for some TCEs hampered by interferences such as the rare-earth elements (REEs) [10]. Recent findings show that the use of nitrous oxide (N_2_O) as reaction gas can lead to even lower detection limits (*x*_L_) compared to O_2_ for some TCEs [11].

Besides the actual measurement by ICP-MS, effective sample digestion is another crucial step in multi-elemental analysis. Closed-vessel microwave-assisted digestion using nitric acid and hydrogen peroxide is a commonly applied technique for plant matrices and comes with many advantages such as automated temperature and pressure control, homogeneous heat transfer, prevention of the loss of volatile elements and minimisation of contamination [12–17]. However, a reduction of recoveries of elements such as Al, Cr, Fe, Ti or the REEs were observed using this mixture dependent on increasing silicon contents (in the form of primary silicates, silicic acids, silica colloids or gels) in the matrix, especially when Si mass fractions exceed 10 mg g^−1^. Traditionally, the dissolvation of silicates is achieved by addition of hydrofluoric acid (HF), which, apart from being highly toxic, can lead to several undesirable effects such as corrosion of analytical instrumentation or the formation of scarcely soluble fluorides of some elements (e.g. Al, Ba, Ca, Cr, Cu, Fe, K, Mg, Mn, Na, Ni, Se, Ti). To prevent these effects, boric acid is frequently added for complexation, which in turn can lead to issues such as increased matrix effects or blank levels. For this reason, alternative approaches including the in situ generation of HF by the use of fluoride-containing chemicals such as tetrafluoroboric acid (HBF_4_) are increasingly investigated [12, 17, 18].

The presented study aims at deploying a fast, easy and robust measurement procedure for the multi-elemental mass fraction measurement in plant samples with a particular focus on the TCEs Li, Be, Ga, Ge, Y, Nb, Sb, the REEs, Ta, Tl and Bi. Particular attention was set on validation of their results with suited certified reference materials (CRMs). Where no certified values in plant CRMs were available (i.e. for Ga, Nb and Ta), spike-recovery experiments were performed to complement the validation. Seven plant CRMs were digested using closed-vessel microwave-assisted digestion with HNO_3_, H_2_O_2_ and HBF_4_ and subsequently analysed with ICP-MS/MS. The CRMs cover a large range of mass fractions and different kinds of plant matrices with varying silicate contents to demonstrate the applicability of the method. The provided data can be used as literature values in future studies and improves the characterisation of commonly used plant CRMs. Apart from the above-mentioned TCEs, elements of common interest were analysed to provide additional information and to show the limitations of the presented measurement procedure.

## Materials and methods

### Reagents and laboratory conditions

All preparatory work except of microwave digestions was performed in an ISO class 8 clean room. Ultra-pure water was obtained from a Milli-Q element module (18.2 MΩ cm; Merck Millipore, Darmstadt, Germany). Nitric acid (HNO_3_, *w* = 65%, p.a. grade; Carl Roth GmbH, Karlsruhe, Germany) and hydrochloric acid (HCl, *w* = 37%, p.a. grade; Carl Roth GmbH) were purified in perfluoralkoxy-polymer (PFA) sub-boiling units (DST-1000 and DST-4000, Savillex, Eden Prairie, MN, USA). Hydrogen peroxide solution (H_2_O_2_, *w* = 30%; Merck KGaA, Darmstadt, Germany) and tetrafluoroboric acid (HBF_4_, *w* = 38%; Chem-Lab EV, Zedelgem, Belgium) to assist digestion were purchased in ultra-pure quality. All plastic consumables were cleaned by soaking in dilute HNO_3_ (*w* = 3%) for at least one day and subsequent thorough rinsing with ultra-pure water. A BL224 BASIC analytical balance (XS instruments, Carpi, Italy) with a readability of 0.01 g and a division of 0.0001 g was used for weighing.

### Calibration standards and CRMs

Calibration standard solutions were prepared gravimetrically. Two 11-point series of calibration standards were prepared, one of them based on the ICP multi-element standard solution VI (Merck Certipur, Darmstadt, Germany) and the second (Nb, Sb and Ta) based on single-element standard solutions. In addition, a 10-point series of calibration standards containing REEs, As, Cr, Fe, Ge, Sb and Se was prepared. For quality control, QC standards were prepared for each series of calibration standards. For elements without certified values in any of the investigated materials (Ga, Nb and Ta), spiked samples were prepared. Further details can be found in the Supplementary information (SI) — Materials and Methods.

The certified reference materials investigated in this study were NIST SRM1515 Apple Leaves and SRM1547 Peach Leaves (National Institute of Standards & Technology, Gaithersburg, Maryland, USA), BCR-129 Hay Powder and BCR-670 Aquatic Plant (European Commission Joint Research Centre, Institute for Reference Materials and Measurements, Geel, Belgium), GBW07603 Bush Twigs and Leaves (also known as NCS DC 73349 Bush Branches and Leaves) and GBW10015 Spinach Leaves (both National Research Centre for Certified Reference Materials, Beijing, China) and NCS ZC73036a Green Tea (NCS Testing Technology Co., Beijing, China).

The selected CRMs cover matrices with varying silicate contents. Silicon is certified in GBW07603 Bush Twigs and Leaves with a mass fraction of 0.60% ± 0.07%, in NCS ZC73036a Green Tea with 0.08% ± 0.01% and in GBW10015 Spinach Leaves with 0.212% ± 0.024%. For BCR-129 Hay Powder, an information value of 2221 µg g^−1^ of Si is given. For NIST SRM1547 Peach Leaves (979 µg g^−1^) and BCR-670 Aquatic Plant (9319 µg g^−1^), no reference values for Si exist. Sucharova and Suchara report 979 µg g^−1^ for SRM1547 and 9319 µg g^−1^ for BCR-670 [12]. For NIST SRM1515 Apple Leaves, no information on the Si content could be found in literature. However, a Si mass fraction of about 30 µg g^−1^ is estimated based on Fig. 4 in Wang et al. 2016, a study conducted on apple leave samples [19]. Thus, the silicate contents in the investigated materials can be ranked as follows: BCR-670 > GBW07603 > BCR-129 > GBW10015 > SRM1547 > ZC73036a > SRM1515.

### Microwave digestion procedure

An Anton Paar Multiwave PRO closed-vessel digestion system equipped with a 24HVT50 rotor (Anton Paar, Graz, Austria) and 30 ml PTFE-vessels was used for microwave digestion of the plant reference materials. Before digestion, three 0.2 g aliquots of each material were taken for determination of the moisture content. The plant material was dried in a drying cabinet at 60 °C for 3 weeks until constant mass was achieved and re-weighed after the drying process. To avoid contamination, these aliquots were not processed further.

For method development, three different reagent mixtures were tested, adapted from literature [12–14, 17]:

A. 5 ml HNO_3_ and 1 ml H_2_O_2_; applied to a subset of 5 CRMs (NIST SRM1547, BCR-129, BCR-670, GBW07603 and GBW10015) in 3 replicates each
B. 4.5 ml HCl, 1.5 ml HNO_3_ and 0.1 ml HBF_4_; applied to NIST SRM1515 in 16 replicates
C. 5 ml HNO_3_, 1 ml H_2_O_2_ and 0.1 ml HBF_4_; applied to all 7 CRMs in 16 replicates each

For each replicate, about 0.2000 g of CRM were weighed into PTFE digestion vessels. After addition of chemicals, the microwave programme was set to a maximum temperature of 200 °C, which was reached within a ramp time of 10 min and then held for 15 min. The maximum microwave power was 1500 W. After cooling to 55 °C, the programme was finished, and the vessels were vented and opened. The digestion vessels were emptied into tared 50 ml PP tubes and rinsed with ultrapure water to obtain a total of about 40 ml of digest. The tubes were weighed empty and after addition of the digest solution to determine the exact masses. Along with each batch, 4 (reagent mixture A) or 8 (B and C) procedural blanks were processed exactly the same way in the microwave. Two cleaning digestions were performed using the same microwave programme with 5 ml of HNO_3_ in each vessel between each batch digested with reagent mixture B or C. The second round of these cleaning digests was collected, diluted and measured in the same way as the CRMs in order to monitor contamination and carry-over effects. Before ICP-MS measurements, the digests were further diluted 1:5 with ultrapure water to achieve an acidity corresponding to *w* = 2% HNO_3_.

### Instrumental procedure

All measurements were performed on a PerkinElmer NexION 5000 ICP-CRC-MS/MS instrument (PerkinElmer, USA) coupled to an ESI SC-2 DX FAST autosampler. Preliminary measurements were conducted on a PerkinElmer NexION 2000 ICP-CRC-MS instrument. The instrumental parameters are given in the SI — Materials and Methods, Table S4. After each sample, the system was rinsed with HNO_3_ (*w* = 3%). To monitor carry-over effects, HNO_3_ analysis blanks (*w* = 2%, to reflect acid mass fractions of diluted samples and calibration standards), were run throughout the measurement at least after every four samples. Quality control solutions were run throughout the analysis at least after every ten samples. To correct for instrumental drift, indium was used as an internal normalisation standard. Isotopes at given *m*/*z* which were affected by spectral interferences were measured on-mass or in mass-shift mode (+ 16) with N_2_O as reaction gas, where most isotopes could be measured without significant interferences. On ^70^Ge^16^O^+^, the ^70^Zn^16^O^+^ interference was corrected by monitoring Zn as ^66^Zn^16^O^+^.

### Data processing

The data was processed using the Syngistix software version 3.1 (PerkinElmer, USA). Limits of detection (*x*_L_) and quantification (*x*_Q_) were calculated once based on the repeatability of the procedural blanks (*n* = 8, mean + 3 × *SD* and mean + 10 × *SD*) and once based on the repeatability of analysis blanks (3 × *SD* and 10 × *SD*). The respective higher results were used as the final *x*_L_ and *x*_Q_. (Note: Because the Syngistix software substracts the signal of an HNO_3_ blank analysed at the start of the measurement automatically from the reported mass fractions, the mean of the analysis blank is not considered for calculation of *x*_L_ and *x*_Q_.) An ESD (Generalized Extreme Studentised Deviate) test was used in order to identify possible outliers. Uncertainties with a coverage factor of 2 (*U*, *k* = 2) were calculated for each element according to the Guide to the Expression of Uncertainty in Measurement (GUM) using a Kragten approach [20]. *Z*-scores and *E*_*n*_ numbers were calculated based on DIN ISO 13528:2015 (E) according to Eqs. 1 and 2 to check for agreement between certified values and the obtained data.

1 $$z=\frac{{x}_{lab}-{X}_{ref}}{\widehat{\sigma }}$$

2 $${E}_{n}=\frac{{x}_{lab}-{X}_{ref}}{\sqrt{{u}_{lab}^{2}+{u}_{ref}^{2}}}$$

*x*_lab_: mean result of this study

*X*_ref_: reference value

$\widehat{\sigma }$: standard deviation for proficiency testing, in this study: *U* given in the certificate

*u*_lab_: combined uncertainty (*U*, *k* = 2)

*u*_ref_: uncertainty of the certified value

*Z*-scores below or equal to 2 and above or equal to  − 2 do not show any significant bias. *E*_*n*_ numbers below or equal to 1 and above or equal to  − 1 indicate satisfactory agreement within the uncertainty of the measurement. The critical values are set closer to 0 for *E*_*n*_ numbers because these are calculated using the expanded uncertainties [21, 22].

### REE data treatment

Rare-earth element signal intensities (including yttrium) were measured in mass-shift mode with N_2_O as reaction gas to circumvent the need for correction of interferences. The REE data was normalised and plotted according to a well-established approach [23, 24]. The most commonly used REE mass fractions for normalisation are data from *chondrite* (for bulk earth or earth’s mantle rocks and most magmatic rocks) and *shale* composite values, e.g. PAAS (post-Archean Australian shale representing the average present day continental crust) for normalising crustal sediments and soils. In this study, the data set of the European shale composite (EUS) was used for normalisation [25]. The elemental REE mass fractions presented are those obtained for the isotope with the best recoveries (i.e. the lowest bias relative to the reference value) or, if recoveries are similar, those with the lowest uncertainties. By normalisation, smooth patterns were obtained that only show positive or negative peaks in case of any anomalies. Anomalies might stem from fractionation due to changes in the redox conditions during (bio)geochemical cycles, but also from anthropogenic impact. In addition, the general smoothness of the curve gives an indication about the quality of the measurement results.

## Results and discussion

### Method development

The digestion efficiency of the 3 tested reagent mixtures A, B and C was compared both visually and considering recoveries. With the use of reagent mixtures A (5 ml HNO_3_ and 1 ml H_2_O_2_) and B (4.5 ml HCl, 1.5 ml HNO_3_ and 0.1 ml HBF_4_), visible residues were found in the digest. In addition, the subsequent ICP-MS measurements delivered lower recoveries for some elements compared to reagent mixture C, e.g. for Li, Na, Al, V, Zn, Ga, Sr, Nb, Cd, Sb, Ba, most REEs, Tl and Pb. This was observed in particular for materials which are expected to have higher contents of Si, such as BCR-670 (according to Sucharova and Suchara [12]). Consequently, mixture C was chosen as digestion procedure. The according data for mixtures A and B can be found in the SI for the respective materials. Cr and Cd, as elements commonly affected by interferences, were measured both in STD mode, in DRC-mode on-mass and mass-shift (+ 16). The obtained biases vary between the investigated materials, but overall, the use of N_2_O did not lead to improved results for these elements. For Cd, a possible explanation is the low oxidation rate with N_2_O which reaches 0.8% at the optimum gas flow. Considering the low Cd levels in plant matrices, other collision/reaction gases would be preferable. For V, the use of HBF_4_ led to an increase in the detection limits due to the ArB^+^ interference on *m*/*z* 51. The analysis on-mass with N_2_O led to both decreased biases relative to the reference values and lower detection limits.

### Results of CRMs

**Fig. 1:**
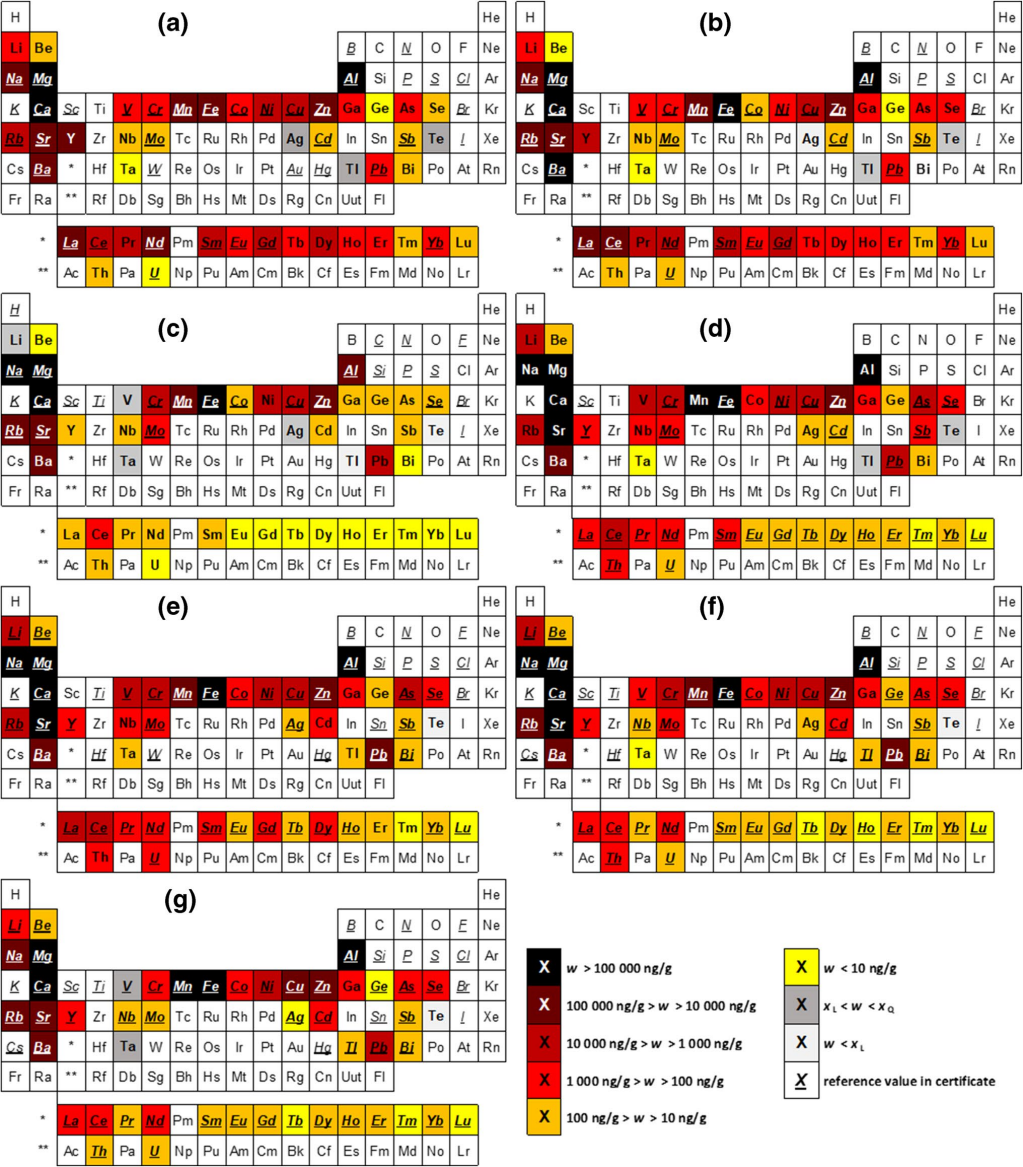
Ranges of contents of 48 analytes in 7 CRMs; **a** NIST SRM1515 Apple Leaves, **b** NIST SRM1547 Peach Leaves, **c** BCR-129 Hay Powder, **d** BCR-670 Aquatic Plant, **e** GBW07603 Bush Leaves and Twigs, **f** GBW10015 Spinach Leaves, **g** NCS ZC73036a Green Tea. Darker shades indicate higher mass fractions

The mean analytical results including combined uncertainties are given in Table 1. The measurement results were corrected for the determined moisture content, which ranged between 5 and 10%. The 48 analytes are grouped into 6 ranges of mass fractions (*w*) on the periodic tables in Fig. 1a–g. The elements occurring in the highest amounts in all 7 CRMs are Mg and Ca with *w* > 100 000 ng g^−1^. Lowest levels were found of Ta with quantifiable mass fractions *w* < 10 ng g^−1^ in all materials except of one (GBW07603: *w*(Ta) = 18 ng g^−1^). The complete measurement results including mass fractions with uncertainties, recoveries, detection and quantification limits for all 48 elements can be found in the SI, Tables SA1–SG3.

**Table 1:**
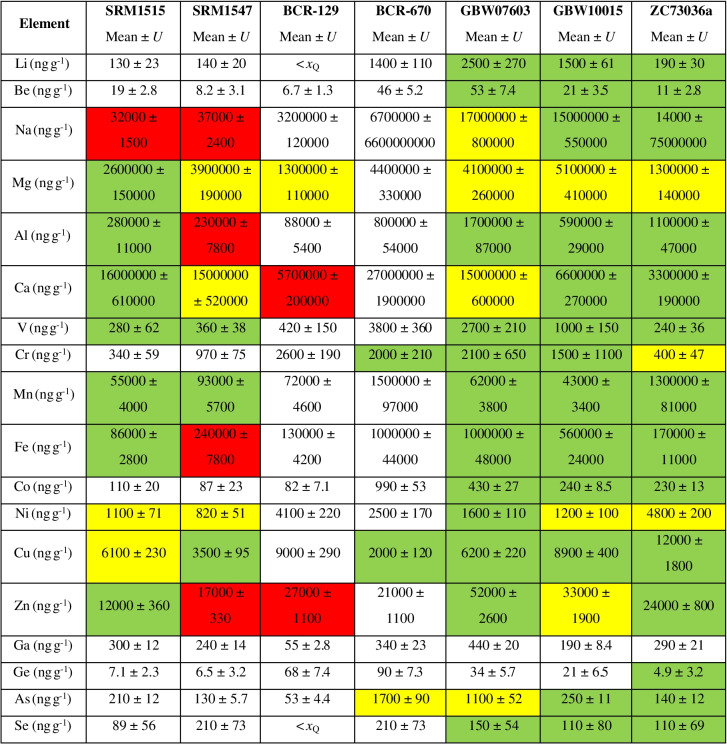

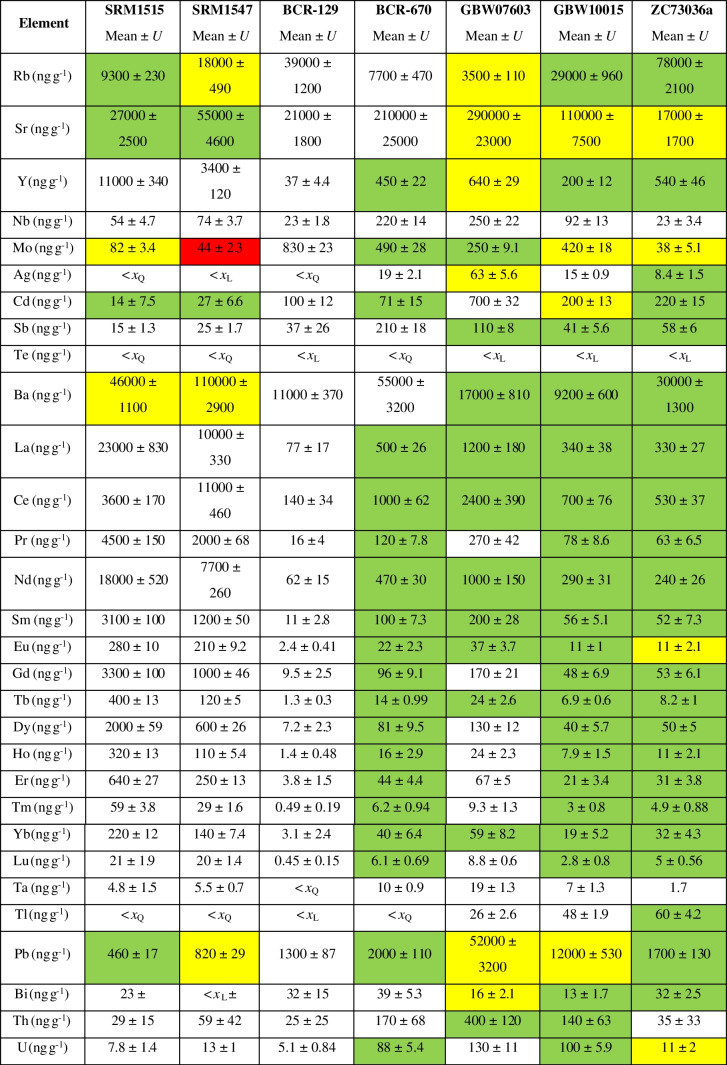
Mean analytical results for 48 analytes in 7 plant CRMs in ng g^−1^. Total combined uncertainties *U,* combining measurement uncertainty and variability between replicates, are given with a coverage factor of 2 (*k* = 2). Results below the limit of detection (quantification) are indicated with  < *x*_L_ (< *x*_Q_). Colours give an indication about closeness of agreement with the certified reference value, where available, by measure of the *E*_n_ numbers. Green: ∣*E*_n_∣ < 1; yellow: 1 < ∣*E*_n_∣ < 2; red: ∣*E*_n_∣ > 2

### Results for 9 selected TCEs

The measurement results for Li, Be, Ga, Ge, Nb, Sb, Ta, Tl and Bi are discussed in more detail in the following. The presented mass fractions are given with combined uncertainties (*U*, *k* = 2).

#### Lithium

The obtained mass fractions for lithium are ranging from *w* = 130 ng g^−1^ ± 23 ng g^−1^ (SRM1515) to 2500 ng g^−1^ ± 270 ng g^−1^ (GBW07603). The element is certified in GBW07603, GBW10015 and ZC73036a. Biases of  − 5.0%, 4.0% and  − 6.7% were obtained, respectively, with related *E*_*n*_ numbers of  − 0.28, 0.25 and  − 0.31, indicating good agreement with the certified values. As Li does not wash out well from the sample introduction system, care must be taken to ensure that carry-over effects do not hamper the analysis. Depending on the levels, prolonged rinsing time or higher acid strength is needed. Samples should be sorted, if possible, according to ascending Li concentrations. If this aspect is considered, Li is not a particularly challenging element to analyse and can be measured without a collision/reaction cell.

#### Beryllium

The determined mass fractions of beryllium in the investigated CRMs lie between *w* = 6.7 ng g^−1^ ± 1.3 ng g^−1^ (BCR-129) and 53 ng g^−1^ ± 7.4 ng g^−1^ (GBW07603). Similarly to Li, it is certified in GBW07603, GBW10015 and ZC73036a, for which 3.5%, 22% and  − 8.3% recovery were obtained, respectively, with *E*_*n*_ numbers of 0.21, 0.92 and  − 0.29.

#### Gallium

For gallium, mass fractions between *w* = 55 ng g^−1^ ± 2.8 ng g^−1^ (BCR-129) and 440 ng g^−1^ ± 20 ng g^−1^ (GBW07603) were found. Ga is not certified in any of the selected CRMs. In previous studies, strongly diverging literature values were reported for SRM1547. A wide range of values between 46 ng g^−1^ [26] and 2.0 µg g^−1^ [27] were found for the material. The Ga mass fraction of 240 ng g^−1^ ± 14 ng g^−1^ determined in this study lies between these extremes. Sucharova and Suchara [12] found Ga mass fractions ranging from 119 to 132 ng g^−1^ in SRM1547 in a broad study testing 5 different digestion procedures. Possible explanations for the large discrepancy between different studies could be incomplete digestion procedures, blank overcorrection or uncorrected interferences at *m*/*z* 71 caused by, e.g. MnO^+^, ArP^+^, ArCl^+^ or Nd^2+^. For the other CRMs, no literature or reference values are available. The strong variation between literature values underlines the urgent need for certified values for this emerging TCE to allow profound method validation. In the present study, the obtained results are validated with spike-recovery experiments performed with SRM1515, BCR-670 and GBW10015, yielding 115% ± 12% recovery.

#### Germanium

Germanium was found in mass fractions between *w* = 4.9 ng g^−1^ ± 3.2 ng g^−1^ (ZC73036a Green Tea on *m*/*z* 72) and 92 ng g^−1^ ± 17 ng g^−1^ (BCR-670 Aquatic Plant on *m*/*z* 73). It is given as information value in GBW10015 Spinach Leaves and certified in ZC73036a. For GBW10015, depending on the measured *m*/*z*, biases between  − 9.0% and 11% were obtained and for ZC73036a, in contrast, biases between  − 34% and  − 5.0%. Due to the high uncertainties of both the certified value (*U* = 1.6 ng g^−1^) and the results obtained in this study (*U* = 3.2 ng g^−1^–7.6 ng g^−1^), the *E*_*n*_ numbers are still low enough (between  − 0.72 and  − 0.24) to prove good agreement with the certificate considering the obtained uncertainty. Unfortunately, there is no certification report available for any of these two materials. The only information about the analytical method available in the certificates is that ICP-MS was used. In personal communication, NCS stated that for the determination of Ge in ZC73036a, both single- and triple-quadrupole instruments were used, both using a collision/reaction cell and standard/no-gas mode. It was further stated that no significant difference could be observed between the obtained results. In our study, single- and triple-quadrupole instruments (NexION 2000 and 5000) were tested for method development. For the single-quadrupole, standard mode, He as collision gas as well as O_2_ and N_2_O as reaction gases were tested. For the triple-quadrupole, only mass-shift mode (+ 16) with N_2_O was tested. In our case, all measured isotopes delivered tremendously different results in the different analysis modes, except from ^74^Ge which varied to a lesser extent due to less prominent interferences. We obtained the best recoveries for ZC73036a using He mode, on *m*/*z* 74. However, due to the good recoveries for GBW10015 and the consistent results for all isotopes, it was decided to choose ICP-MS/MS in mass-shift mode with N_2_O as reaction gas for the final method. In this way, it was possible to measure the isotopes ^72^Ge, ^73^Ge and ^74^Ge without significant interferences. The isobaric interference of ^74^Se on ^74^Ge is negligible due to the low abundance of that isotope in combination with the low oxidation rate of Se with N_2_O at the chosen flow rate of 0.4 mL min^−1^. ^70^Zn was corrected mathematically based on the ^68^Zn signal, as stated above under “Instrumental procedure” section.

#### Niobium

The mass fractions obtained for niobium in the 7 CRMs range from *w* = 23 ng g^−1^ ± 1.5 ng g^−1^ (ZC73036a) to 250 ng g^−1^ ± 22 ng g^−1^ (GBW07603). Nb is not certified in any of the investigated CRMs; however, it is given as information value in GBW10015 and ZC73036a. The biases obtained in this study were 53% in both cases. The apparent overestimation can be explained with the high uncertainties on information values. Spike-recovery experiments performed with SRM1515, BCR-670 and GBW10015 resulted in a mean recovery of 97% ± 20%.

#### Antimony

Antimony mass fractions range from *w* = 14 ng g^−1^ ± 1.8 ng g^−1^ (SRM1515 Apple Leaves on *m/z* 123) to 220 ng g^−1^ ± 16 ng g^−1^ (BCR-670 Aquatic Plant on *m/z* 123). Certified or information values are given for Sb in all investigated CRMs except from BCR-129 Hay Powder. Biases were within  − 5.1% and 25%, with *E*_*n*_ numbers for the certified values ranging from 0.33 to 1.0. Extreme outliers with values about three times as high as the mean of the remaining replicates were eliminated in some of the CRMs by using ESD tests (4 in SRM1515, 3 in BCR-129, 1 in GBW07603 and 2 in GBW10015). As the cleaning digests between each digestion batch were collected and analysed, contamination during the sample preparation procedure can be ruled out as a possible source. In case of BCR-129, a correlation with tellurium could be observed, which would be significant if Te mass fractions were not below *x*_L_ in the material. A correlation would indicate the presence of nuggets within the material containing both elements and indicating heterogeneity of the material. For the other elements, no significant correlations were observed. As Sb will never appear as pure nuggets in nature, other elements might be elevated as well; however, the absolute differences might be too small to identify correlations with more abundant elements such as copper.

#### Tantalum

For tantalum, mass fractions between *w* = 4.8 ng g^−1^ ± 1.5 ng g^−1^ (SRM1515) and 19 ng g^−1^ ± 1.3 ng g^−1^ (GBW07603) were determined. There are no reference values available in the certificates of the selected plant CRMs. For SRM1547, Rodushkin et al. [26] report 7 ng g^−1^ ± 2 ng g^−1^, whereas the result obtained in the present study was 5.5 ng g^−1^ ± 0.70 ng g^−1^. Ta results were validated with spike-recovery experiments for which an average recovery of 97% ± 23% was obtained.

#### Thallium

Thallium was found in a range of *w* = 26 ng g^−1^ ± 2.6 ng g^−1^ (GBW07603) to 60 ng g^−1^ ± 4.2 ng g^−1^ (ZC73036a). The mass fraction of the element is certified in ZC73036a. The measurement results show a bias of 5.6% and an *E*_*n*_ number of 0.27 in the material. In addition, an information value is given in GBW10015, for which a bias of -1.2% was obtained in this study. The *x*_L_ (see SI, Tables SA3–SG3) for Tl is increased due to the large scatter of the procedural blank results, which is the reason why the element could be quantified in only three out of the seven materials (apart from the two above mentioned, in GBW10015). This element is, similarly to Li, an element which is known to cause significant carry-over effects in the sample introduction system of ICP-MS systems [28]. This property might have led to elevated blank levels in some of the microwave digestion vessels. In each batch, among the 8 method blanks, 4 were elevated about one order of magnitude. Before the experiments for this study, they were used for samples, while the others had always been used for blanks only. In the cleaning digests between the batches, Tl backgrounds seemed to be distributed evenly across all vessels. Since the cleaning was performed with HNO_3_ only, whereas the sample digestion also contained H_2_O_2_ and HBF_4_, it appears that one of these chemicals, presumably HBF_4_, enhanced leaching of Tl. For future low-level analysis of Tl, special care must be taken to prevent such carry-over effects by cleaning the digestion vessels, if possible, with the same chemicals as used for sample digestion.

#### Bismuth

Bismuth was found in levels between *w* = 13 ng g^−1^ ± 1.7 ng g^−1^ (GBW10015) and 39 ng g^−1^ ± 5.3 ng g^−1^ (BCR-670). Certified reference values are available for GBW10015, ZC73036a and GBW07603. The biases obtained in this study were  − 0.49%, 3.6% and  − 30%, respectively, with related *E*_*n*_ numbers of  − 0.034, 0.20 and − 1.3.

### Rare-earth elements

Among the CRMs investigated in this study, there are three for which certified values including an uncertainty are available for all REEs: BCR-670 Aquatic Plant, GBW10015 Spinach Leaves and NCS ZC73036a Green Tea. For SRM1515, SRM1547, BCR-670 and GBW07603, data from previous studies is plotted in addition to the values given in the certificate for comparison [14, 26, 29, 30]. For BCR-129 Hay Powder, no REE reference data is available.

**Fig. 2:**
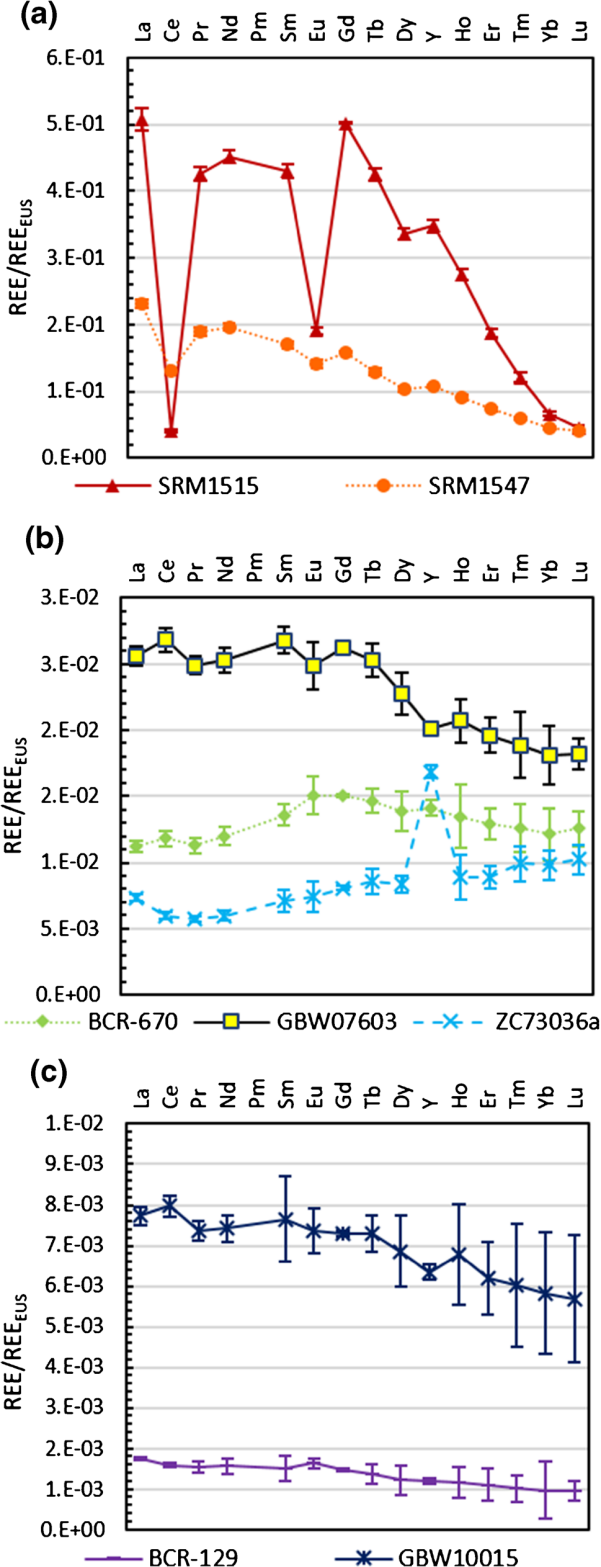
EUS-normalised REE patterns of 7 plant CRMs. Error bars indicate measurement uncertainties based on a single replicate

**Fig. 3:**
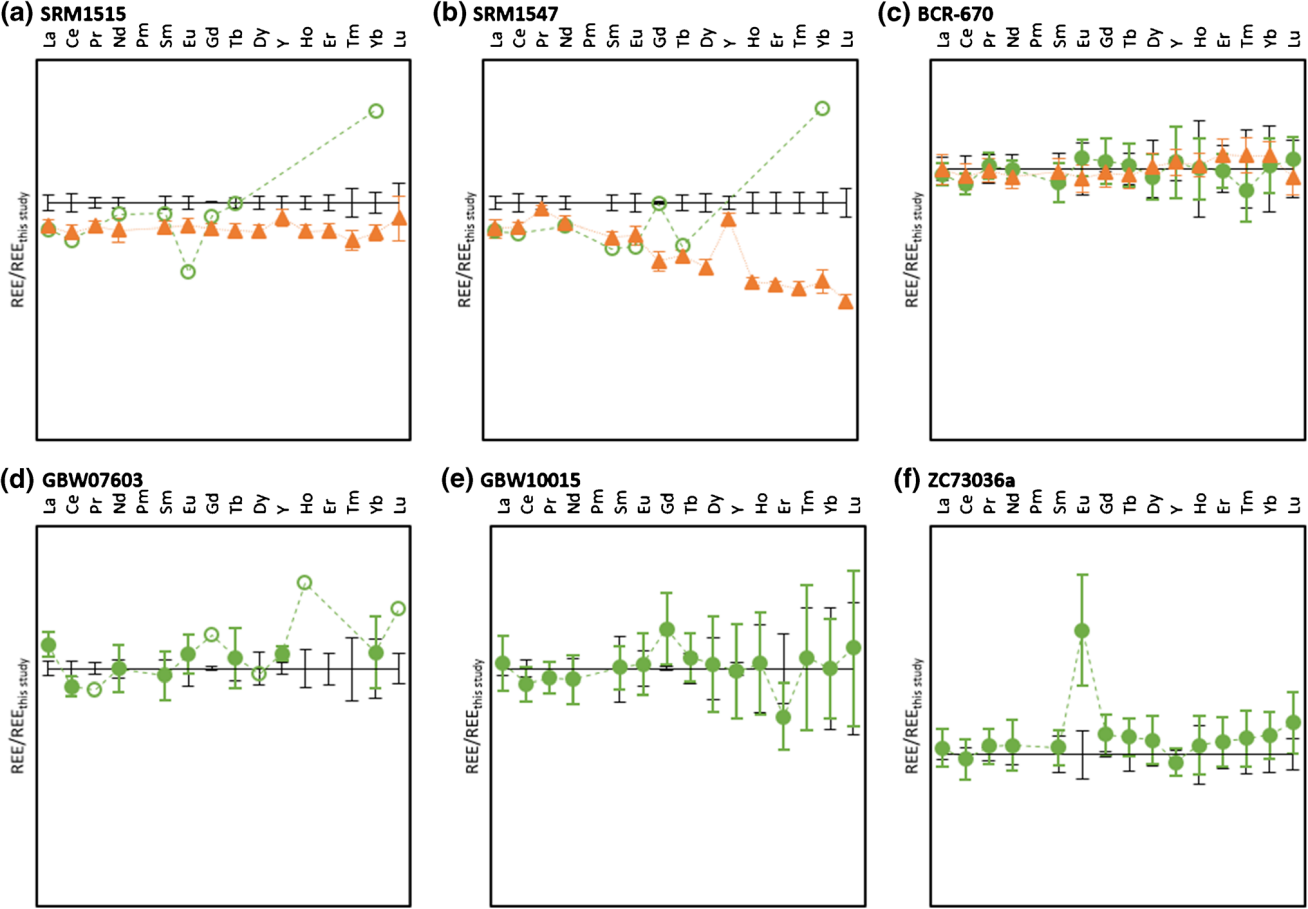
Literature data (triangles with dotted line [14, 26, 29]) and reference values (circles with dashed line; full circles for certified values, empty circles for information values) normalised to data obtained in this study (straight black lines, with error bars indicating measurement uncertainties based on a single replicate)

The patterns obtained from REE data normalised to European shale composite (REE_EUS_) are shown with their respective measurement uncertainties (*U*_meas_ based on a single replicate, *k* = 2%, confidence interval 95%) in Fig. 2a-c. The data along with combined uncertainties (*U*) can be found in the SI. From the graphs, it can be seen that uncertainties increase with decreasing REE contents, which has to be taken into account when interpreting the data and choosing the right CRM for the validation of a measurement procedure. In Fig. 3a-f, literature data and reference values are normalised to data from this study for comparison.

#### NIST SRM1515 Apple Leaves

The EUS-normalised REE data for NIST SRM1515 Apple Leaves is shown in Fig. 2a, whereas a comparison of data from this study to reference values and literature data can be found in Fig. 3a. For the most redox-sensitive REEs Ce and Eu, strong negative anomalies are observed both in our data, in a previous study by Bandoniene et al. [29] and in the reference values. In sediments and soils, Eu and Ce anomalies indicate changes in the redox conditions. While Eu is removed in reducing conditions, Ce is removed in oxidising conditions. Depending on the species, however, plants do not always reflect the REE patterns of the parent soil, as also discussed in literature [29]. The observed anomalies might therefore be intrinsic features of the plant material. The biases for the elements for which information values are available (La, Ce, Nd, Sm, Eu, Gd, Tb, Yb) are within 0.31% and 18% in SRM1515, except for Eu (41%) and Yb (-28%). As the analytical method for the determination of information values is not described in further detail by NIST, the discrepancies cannot easily be explained. However, our values are in good agreement with those of Bandoniene et al. [29] and give a smooth pattern for heavy rare-earth elements (HREE) except for a slight Y/Ho fractionation. This observation is, again, shared by Bandoniene et al. [29].

#### NIST SRM1547 Peach Leaves

The normalised REE pattern for the second NIST material investigated in this study, SRM1547 Peach Leaves, is shown in Fig. 2a. Literature data and reference values normalised to data from this study can be seen in Fig. 3b. Similarly to SRM1515, our values again give a smooth pattern for HREEs except from an apparent slight Y/Ho-fractionation, which is found to an even larger extent by Roduskin et al. [26]. The biases for SRM1547 range from 0.57 to 24%, with the exception of Yb (-30%) which seems to be underestimated to the same extent as in SRM1515 in relation to the information value stated in the certificate.

#### BCR-129 Hay Powder

BCR-129 Hay Powder is, among the investigated CRMs, the material with the lowest contents of REEs (low ng g^−1^ range) and thus with the highest measurement uncertainties. To our knowledge, no literature data is available for REEs in BCR-129. The plot of the EUS-normalised data is shown in Fig. 2c. In general, the pattern appears smooth. Due to the high uncertainties on the results for this material, the presented results can only serve as a rough indication of the contained levels.

#### BCR-670 Aquatic Plant

For BCR-670 Aquatic Plant, certified values including uncertainties for all REEs and a comprehensive certification report are available. The values obtained in this study are in good agreement with the certified values (− 3.7 to 8.2% bias). A previous study conducted by Zocher et al. [14] compared a high-pressure high-temperature decomposition protocol using HNO_3_, HCl and HF to a low-pressure low-temperature extraction protocol using HNO_3_ only. In Fig. 3c, the data obtained for the protocol using HNO_3_, HCl and HF followed by preconcentration and matrix separation is displayed along with the certified values, both normalised to the data from our study. Considering the full combined uncertainties, no significant differences between the curves can be observed, which is also depicted in the *E*_*n*_ numbers (− 0.36 to 0.80). When comparing the error bars of the data displayed in Fig. 3c, it has to be considered that the data from our study is shown with measurement uncertainties, while Zocher et al. [14] present their data with the RSDs of the mean of the digestion replicates (*n* = 13). Our replicate RSDs range from 1.3 to 3.0% (*n* = 15–16) compared to those from Zocher et al. [14] which lie between 4.2 and 6.8%. Therefore, we consider our method to deliver data of equal quality. In this way, it is shown that mathematical corrections, which were carefully applied in the other study, preconcentration and/or matrix separation are not needed for matrices similar to BCR-670 when analysing REEs in mass-shift mode with ICP-MS/MS.

#### GBW07603 Bush Twigs and Leaves

The EUS-normalised data obtained for GBW07603 Bush Twigs and Leaves is shown in Fig. 2b. Biases for certified reference values range from  − 7.7 to 10%, for information values from  − 27 to 12%. Upon visual comparison in Fig. 3d, the reference values do not seem to be in good agreement with the ones derived from our data. However, the *E*_*n*_ numbers calculated for the certified values (La, Ce, Nd, Sm, Eu, Tb, Y and Yb) all lie between  − 1 and 1 indicating no significant differences within the uncertainties. To our knowledge, the present study is the first one to report mass fractions for the full set of REEs in this material. Because of the smoothness of the curve based on our data and the unlikeliness of Ho-enrichment in the plant material, we consider our data reliable.

#### GBW10015 Spinach Leaves

For GBW10015 Spinach Leaves, certified values including uncertainties are available for all REEs including Y. Our biases range from  − 5.7 to 26%, and all obtained mass fractions result in an *E*_*n*_ number between  − 1 and 1. The reference values seem to indicate a slight positive Gd anomaly and negative Er anomaly (Fig. 3e). This is not confirmed by our own data, which gives a smooth pattern (Fig. 2c). In general, positive Gd anomalies occur e.g. through anthropogenic contamination with MR contrasting agents, but also through spectral interferences, e.g. from Ce and Pr (CeO^+^, CeOH^+^ and PrO^+^) [31]. Considering the obtained *E*_*n*_ numbers, the bias is not significant.

#### NCS ZC73026a Green Tea

The last CRM investigated in this study is NCS ZC73036a Green Tea. The certified value for Eu deviates strongly from the one obtained in this study (− 45% bias). With an *E*_*n*_ number of  − 2.0, this difference can be considered significant. Looking at the normalised pattern in Fig. 3f, the certified values would indicate a positive Eu anomaly. A possible explanation for that would be an uncorrected BaO interference (^135^Ba^16^O^+^ on ^151^Eu^+^ and ^137^Ba^16^O^+^ on ^153^Eu^+^) on the certified value, which was eliminated in the present study by analysing EuO in mass-shift mode. Since the only information about the analytical methodology available for ZC73036a is that ICP-MS was used, this cannot be confirmed. In contrast, the strongly disturbed natural ratio of Y and Ho indicated by the reference values was confirmed in the present study. It might origin from the use of zirconium oxide ceramics for milling of the reference material which typically contain some Y content for purpose of stabilisation. Considering that a CRM is simply defined as a homogenous material which may also contain contamination of non-natural origin as long as these are distributed evenly, this would not hamper the suitability of the CRM for the purpose of method validation.

## Conclusions

An optimised measurement procedure for the analysis of 48 elements including TCEs in plants is described, and its validity is discussed. The presented results are in good agreement with the available reference values as proven by the *E*_*n*_ numbers. It was shown that the use of HBF_4_ leads to improved recoveries for elements such as Li, Na, Al, V, Zn, Ga, Sr, Nb, Cd, Sb, Ba, most REEs, Tl and Pb, especially for plant matrices which can be expected to have higher silicate contents (reflected by BCR-670 and GBW07603). Compared to conventional methods, which often require extensive mathematical correction associated with large uncertainties, pre-concentration and matrix separation in order to deliver valid results, the ICP-MS/MS technique offers a convenient way for significant reduction of interferences for Ge and the REEs without any additional steps in sample preparation or data treatment after measurement. With the reduced amount of time and resources needed per sample, larger sets of samples can be analysed, which will be of great benefit for environmental monitoring of possible emerging contaminants in low levels.

In the presented study, 48 elements were analysed in 7 plant CRMs with a particular focus on TCEs. For these elements, only a small number is certified, and only scarce additional literature information is available so far. The presented data leads to a significant extension of the information content of commonly used CRMs. For future research about environmental levels of TCEs, more CRMs are needed for plant matrices. This is especially the case for the elements Ga, Te and Ta, where to the knowledge of the authors, no plant CRMs exist so far. Reliable reference data is also very scarce for Ge, Nb and Tl. Considering the tremendously increased use of individual TCEs and thus the possibility of higher environmental background levels, as well as the challenging analysis for some of them, priority should be set on the production and characterisation of CRMs for TCEs in plants.

## Supplementary Information

Below is the link to the electronic supplementary material.

Supplementary file1 (DOCX 32 KB)

Supplementary file2 (XLSX 188 KB)
