# Supplementary material for: Determination of 48 elements in 7 plant CRMs by ICP-MS/MS with a focus on technology-critical elements
Source: Anal Bioanal Chem. 2023 Jan 10;415(6):1159–72. doi: 10.1007/s00216-022-04497-3 (PMC9899746; doi:10.1007/s00216-022-04497-3)
Supplement: Supplementary file 1 — Supplementary file1 (DOCX 32 KB) [file 216_2022_4497_MOESM1_ESM.docx]

**Supplementary Information – Materials and Methods**

**Determination of 48 elements in 7 plant CRMs by ICP-MS/MS with a focus on technology-critical elements**

Simone Trimmel, Thomas C. Meisel, Shaun T. Lancaster, Thomas Prohaska, Johanna Irrgeher*
Chair of General and Analytical Chemistry, Montanuniversität Leoben, Leoben, Austria
*Correspondence: : [johanna.irrgeher@unileoben.ac.at](mailto:simone.trimmel@unileoben.ac.at)

ORCID

Simone Trimmel: 0000-0002-2102-5392

Thomas Meisel: 0000-0001-5572-3212

Shaun T. Lancaster: 0000-0001-8843-6893

Thomas Prohaska: 0000-0001-9367-8141

Johanna Irrgeher: 0000-0003-3192-0101

## Calibration standards and CRMs

Calibration standard solutions were prepared gravimetrically. For the elements analysed in standard mode, 2 series of calibration standards were prepared. The ICP multi-element standard solution VI (Merck Certipur, Darmstadt, Germany) is in the following referred to as “MVI” and contains 989 mg L^−1^ of Ca; 102 mg L^−1^ of Be; 100 mg L^−1^ of Fe; 99 mg L^−1^ of Zn; 10 mg L^−1^ of Ag and Sr; 9.9 mg L^−1^ of Cd, Co, Cr, Cu, Ga, Li, Mn, Mo, Ni, Rb, Te and Tl; 9.8 mg L^−1^ of Al, Bi, Mg, Pb, U and V and 9.7 mg L^−1^ of Ba and Na. The stock solution was diluted to obtain an 11-point calibration ranging from (nominal) 0.005 to 50 ng g^−1^ Li. The specification of traceability of the respective elements can be found in Table S1. The other standard solutions from Merck Certipur, are stated to be traceable to NIST, without providing details. For the other standard solutions, no statement regarding traceability is given.

Table S1: Specification of traceability for the elements contained in ICP multi-element solution VI (Merck Certipur)

| **Element** | **NIST Standard Reference Material** |
| --- | --- |
| Ag | SRM 3151 |
| Al | SRM 3101a |
| As | SRM 3103a |
| B | SRM 3107 |
| Ba | SRM 3104a |
| Be | SRM 3105a |
| Bi | SRM 3106 |
| Ca | SRM 3109a |
| Cd | SRM 3108 |
| Co | SRM 3113 |
| Cr | SRM 3112a |
| Cu | SRM 3114 |
| Fe | SRM 3126a |
| Ga | SRM 3119a |
| K | SRM 3141a |
| Li | SRM 3129a |
| Mg | SRM 3131a |
| Mn | SRM 3132 |
| Mo | SRM 3134 |
| Na | SRM 3152a |
| Ni | SRM 3136 |
| Pb | SRM 3128 |
| Rb | SRM 3145a |
| Se | SRM 3149 |
| Sr | SRM 3153a |
| Te | SRM 3156 |
| Tl | SRM 3158 |
| U | SRM 3164 |
| V | SRM 3165 |
| Zn | SRM 3168a |

For the elements analysed with N_2_O, a series of calibration standards containing REEs, As, Cr, Fe, Ge, Sb and Se was prepared. For this purpose, single-element standard solutions of As 1001 ± 5 µg mL^−1^ (Inorganic Ventures, New Jersey, USA), Cr 1000 µg mL^−1^ (Alfa Aesar, Kandel, Germany), Fe 1002 ± 4 µg mL^−1^ (Inorganic Ventures, New Jersey, USA), Se 1000 ± 3 µg mL^−1^ (CPI International, Santa Rosa, USA), Ge and Sb (same as above) as well as the custom-made multi-element standard AHF CAL-7 (Inorganic Ventures, New Jersey, USA) were used. AHF CAL-7 contains the following mass fractions of REEs: 1000 µg mL^−1^ of Ce; 500 µg mL^−1^ of La, Nd, Y; 100 µg mL^−1^ of Pr; 150 µg mL^−1^ of Th; 50 µg mL^−1^ of Dy, Gd, Sm, U; 20 µg mL^−1^ of Er, Eu, Yb; 10 µg mL^−1^ of Ho, Tb; and 5 µg mL^−1^ of Lu, Tm. The stock solutions were mixed and diluted to obtain a 10-point calibration with the ranges 0.005-7.5 ng g^−1^ As, 0.0025-2.5 ng g^−1^ Cr, 0.5-450 ng g^−1^ Fe, 0.0075-0.8 ng g^−1^ Ge, 0.001-1 ng g^−1^ Sb, 0.005-5 ng g^−1^ Se and 0.0005-0.5 ng g^−1^ Er.

For Nb, Sb and Ta, a multi-element standard solution containing 10 µg g^−1^ Ge, Nb and Sb and 0.1 µg g^−1^ Ta was prepared from the single-element standard solutions Ge 1000 µg ml^−1^ (High-Purity Standards, North Charleston, South Carolina, USA), Nb 1000 ± 5 µg ml^−1^ (Inorganic Ventures, New Jersey, USA), Sb 1006 ± 5 µg ml^−1^ (Inorganic Ventures) and Ta 998 ± 4 µg ml^−1^ (Inorganic Ventures). Based on this solution, an 11-point calibration series ranging from 0.001 to 1 ng g^−1^ Ge. A 650 pg g^−1^ In internal standard solution was prepared from 1000 mg L^−1^ single-element standard solution (Merck KGaA, Darmstadt, Germany).

For check of calibration and possible drift, 3 quality control (QC) solutions were prepared. For the elements contained in the MVI calibration standards, a mixture of single-element standard solutions of Na 100 mg L^−1^ (Merck KGaA, Darmstadt, Germany), Mg 1000 mg L^−1^ (Merck KGaA, Darmstadt, Germany), Al 1001 ± 5 µg mL^−1^ (Inorganic Ventures, New Jersey, USA), K 1000 mg L^−1^ (Merck KGaA, Darmstadt, Germany), Ca 1000 mg L^−1^ (Merck KGaA, Darmstadt, Germany), Mn 999 ± 4 µg mL^−1^ (Inorganic Ventures, New Jersey, USA), Fe 1002 ± 4 µg mL^−1^  (Inorganic Ventures, New Jersey, USA), Zn 999 ± 4 µg mL^−1^ (Inorganic Ventures, New Jersey, USA), Se 1000 ± 3 µg mL^−1^ (CPI International, Santa Rosa, USA), Rb 1000 mg L^−1^ (Merck KGaA, Darmstadt, Germany) and Ba 996 ± 4 µg mL^−1^ (Inorganic Ventures, New Jersey, USA) was spiked to a MVI calibration standard. For Ge, Nb, Sb and Ta, a QC standard was prepared from the same stock solutions used to prepare the calibration standards. For the elements analysed with N_2_O, a QC standard was prepared from the multi-element standard “Multi-element Solution 1” (Spex Certiprep, New Jersey, USA) containing 10 µg mL^−1^ of Ce, Dy, Er, Eu, Gd, Ho, La, Lu, Nd, Pr, Sc, Sm, Tb, Th, Tm, Y and Yb, the MVI multi-element standard solution and the single-element standard solutions Ge and Sb (same as above). The mass fractions contained in the QC solutions are given in Table S2 and Table S3.

Table S2: Mass fractions of the analytes contained in the QC solutions used for the runs in standard mode

| Analyte | Mass fraction [ng g^-1^] |
| --- | --- |
| Ta | 0.0030 |
| Ge, Nb, Sb | 0.25 |
| Li, V, Cr, Co, Ni, Cu, Ga, Mo, Ag, Cd, Te, Tl, Pb, Bi, U | 2.8 |
| Rb, Sr | 22 |
| Be, As | 29 |
| Na, Al | 40 |
| Ba | 51 |
| Zn | 56 |
| Mn | 70 |
| Se | 78 |
| Mg | 100 |
| Fe | 140 |
| Ca | 850 |

Table S3: Mass fractions of the analytes contained in the QC solutions used for the runs in N_2_O mode

| Analyte | Mass fraction [ng g^-1^] |
| --- | --- |
| Cr | 0.065 |
| Ge | 0.10 |
| Y, Sb, La, Ce, Pr, Nd, Sm, Eu, Tb, Gd, Dy, Ho, Er, Tm, Yb, Lu, Th, U | 0.10 |
| Fe | 1.0 |
| Se | 1.6 |
| As | 1.7 |

## Spike-recovery experiments

Spiked samples were prepared for Ga, Nb and Ta, as none of these elements is certified in any of the investigated CRMs. For this purpose, diluted digests (prepared as described under 2.3 in the manuscript) of SRM1515, GBW10015 and BCR-670 to reflect low, medium and high silicate content were spiked with either single-element standard solution Ga 10.00 ± 0.06 µg ml^-1^ (Elemental Scientific, USA), 1000 ± 5 µg ml^-1^ Nb (Inorganic Ventures, USA) or 998 Ta ± 4 µg ml^-1^ (Inorganic Ventures). For Ga and Nb, 100, 200 and 300 ng g^-1^ were spiked, for Ta, 0.05, 0.1 and 0.15 ng g^-1^. The spiked mass fractions were chosen to obtain approximately 150 %, 200 % and 250 % of the original mass fractions. The exact spiked masses were determined gravimetrically.

## Instrumental parameters

The instrumental parameters for all measurements performed on the NexION 5000 (PerkinElmer, USA) are given in Table S4.

Table S4: Instrumental parameters

| Parameter | Standard mode | Mass-shift mode |
| --- | --- | --- |
| Analytes | MVI, Nb, Sb, Ta | REE, As, Fe, Ge, Sb, Se, Zn |
| Cell gas | None | N_2_O |
| Cell gas flow | - | 0.4-0.7 mL min^-1^ |
| Spray chamber temperature | 5 °C | 5 °C |
| Interface cones | Nickel | Nickel |
| Nebulizer | PFA-ST-40 44296 | PFA-ST-40 44296 |
| Nebulizer gas flow | 0.97-0.99 mL min^-1^ | 0.97-0.99 mL min^-1^ |
| RF power | 1600 W | 1600 W |
| Plasma gas flow | 16 L min^-1^ | 16 L min^-1^ |
| Auxiliary gas flow | 1.2 L min^-1^ | 1.2 L min^-1^ |
| Data acquisition mode | 6 sweeps/reading, 1 reading/replicate, 6 replicates | 6 sweeps/reading, 1 reading/replicate, 6 replicates |
| Dwell time per replicate | 25-150 ms | 50 ms |
| Integration time | 150-900 ms | 300 ms |
| RPa | 0-0.02 V | 0 V |
| RPq | 0.25 V | 0.45 V |
| Total time/sample | 2 min 13 s | 1 min 39 s |
